# ‘If an Eye Is Washed Properly, It Means It Would See Clearly’: A Mixed Methods Study of Face Washing Knowledge, Attitudes, and Behaviors in Rural Ethiopia

**DOI:** 10.1371/journal.pntd.0005099

**Published:** 2016-10-27

**Authors:** Kristen Aiemjoy, Nicole E. Stoller, Sintayehu Gebresillasie, Ayalew Shiferaw, Zerihun Tadesse, Tegene Sewnet, Bezuayehu Ayele, Melsew Chanyalew, Kelly Callahan, Aisha Stewart, Paul M. Emerson, Thomas M. Lietman, Jeremy D. Keenan, Catherine E. Oldenburg

**Affiliations:** 1 Francis I. Proctor Foundation, University of California, San Francisco, United States of America; 2 The Carter Center, Addis Ababa, Ethiopia; 3 Regional Health Bureau, Addis Ababa, Ethiopia; 4 The Carter Center, Atlanta, GA, United States of America; 5 Department of Ophthalmology, University of California, San Francisco, United States of America; 6 Department of Epidemiology and Biostatistics, University of California, San Francisco, United States of America; 7 Department of Epidemiology, Harvard T.H. Chan School of Public Health, Boston, MA, United States of America; University of California San Diego School of Medicine, UNITED STATES

## Abstract

**Background:**

Face cleanliness is a core component of the SAFE (Surgery, Antibiotics, Facial cleanliness, and Environmental improvements) strategy for trachoma control. Understanding knowledge, attitudes, and behaviors related to face washing may be helpful for designing effective interventions for improving facial cleanliness.

**Methods:**

In April 2014, a mixed methods study including focus groups and a quantitative cross-sectional study was conducted in the East Gojjam zone of the Amhara region of Ethiopia. Participants were asked about face washing practices, motivations for face washing, use of soap (which may reduce bacterial load), and fly control strategies.

**Results:**

Overall, both knowledge and reported practice of face washing was high. Participants reported they knew that washing their own face and their children’s faces daily was important for hygiene and infection control. Although participants reported high knowledge of the importance of soap for face washing, quantitative data revealed strong variations by community in the use of soap for face washing, ranging from 4.4% to 82.2% of households reporting using soap for face washing. Cost and forgetfulness were cited as barriers to the use of soap for face washing. Keeping flies from landing on children was a commonly cited motivator for regular face washing, as was trachoma prevention.

**Conclusions:**

Interventions aiming to improve facial cleanliness for trachoma prevention should focus on habit formation (to address forgetfulness) and address barriers to the use of soap, such as reducing cost. Interventions that focus solely on improving knowledge may not be effective for changing face-washing behaviors.

## Author Summary

Facial cleanliness is a core component of the SAFE (Surgery, Antibiotics, Facial cleanliness, and Environmental improvements) strategy for trachoma control. We conducted a mixed methods study in a trachoma hyperendemic region of rural Ethiopia to better understand knowledge, attitudes, and behaviors related to face washing. Overall, knowledge of the benefits of face washing was high, and participants reported regularly engaging in face washing practices. However, the use of soap for face washing varied more between communities. Participants cited cost and forgetting to use soap as the primary barriers to using soap for face washing. Trachoma prevention, including keeping flies from landing on children’s faces, was a commonly-cited motivator for face washing discussed in focus groups. Given the near-universal knowledge of the benefits of face washing, interventions focused on changing face washing behavior for trachoma control should focus on habit formation and removal of barriers to the use of soap rather than simply educational interventions.

## Introduction

Trachoma is the leading cause of infectious blindness globally.[1–3] Caused by the bacterium *Chlamydia trachomatis*, trachoma is thought to be transmitted by direct contact from infected persons and clothing, as well as the moisture-seeking fly *Musca sorbens*.[4,5] Currently endemic in 53 countries[6], trachoma is estimated to result in blindness or severe vision loss in more than 2 million people[1], with the majority of cases found in sub-Saharan Africa.[1] Despite large reductions in the burden of trachoma in the past several decades[1], trachoma remains an important cause of blindness primarily among individuals living in poor, predominantly rural areas.[6–9]

The cornerstone of trachoma control is the SAFE (Surgery, Antibiotics, Facial cleanliness, and Environmental improvements) strategy.[6] Mass antibiotic distributions have been shown to be effective at reducing the prevalence of trachoma.[10,11] However, while antibiotics may lead to local control of trachoma, alone they may not be sufficient for trachoma elimination in places with hyperendemic infection.[11] Multiple observational studies have demonstrated an association between poor facial hygiene, including the presence of flies on a child’s face, and trachoma.[12–16] It is possible that improvements in hygiene, and especially facial hygiene, may alter the transmission dynamics of trachoma and create more favorable conditions for trachoma elimination.

The use of soap for face washing has been shown to be associated with decreased risk of trachoma in some[16–19] but not all[20] studies. Soap may decrease the bacterial load on children’s faces, which could decrease the probability of transmission of trachoma. A recent meta-analysis of observational studies demonstrated that use of soap was associated with a lower prevalence of trachoma.[16] However, soap specifically for face washing is rarely included or advocated for in trachoma elimination campaigns.

The association between poor facial hygiene and trachoma suggests that interventions to promote facial cleanliness may be helpful in reducing trachoma prevalence and ultimately achieving trachoma elimination. These interventions will benefit from understanding current knowledge, practices and beliefs related to face washing. Here, we analyze knowledge, beliefs, and practices related to face washing, and their relation to trachoma, in a mixed methods study in a trachoma-hyperendemic region of rural Ethiopia.

## Methods

### Study context

This study took place in a rural agrarian region in the Goncha Siso Enese *woreda* of East Gojjam, Amhara, Ethiopia. The communities in this study were participating in a series of cluster-randomized trials testing different mass drug administration strategies for trachoma elimination beginning in 2006. Each community has approximately 275 residents. These communities received mass azithromycin distributions annually or biannually between 2006 and 2013.[11] Methods for these trials are described in detail elsewhere.[11] At baseline, the prevalence of trachoma in children 1–10 years old was 48.5% and 15.5% in children 11 years and older.[11] For the present study, we selected five communities that were within a one-hour walk from the farthest place a four-wheel drive vehicle could reach. All households in each community included in this study were eligible to participate in the quantitative survey. Before and during the study, all communities continued to receive the prescribed government package of hygiene promotion activities. In this report, the five communities are labeled Community A, B, C, D, and E to protect anonymity of the communities.

The quantitative and qualitative surveys were designed to gain an understanding of existing knowledge and behaviors in relation to face washing, and to identify gaps between knowledge and behaviors.

#### Qualitative phase

In April 2014, focus groups were conducted in each of the five communities. Three separate focus groups with seven members each were conducted in each community, including one with exclusively female participants, one with exclusively male participants, and one with female and male community leaders, for a total of 15 focus groups. Community health workers used convenience sampling to enroll focus group by approaching households and asking for volunteers until the group had seven participants. Community leaders were recruited from the local government and from the health development army. The health development army is a group of community members who have been recruited by the local government to assist with implementation of development activities in Ethiopia. The health development army volunteers receive regular training from government-employed health extension workers. The focus group guides were developed based on the United Nations Children’s Emergency Fund (UNICEF) technical manual on hygiene promotion, and included items related to knowledge and perceptions about hygiene (e.g., participants’ definition for what hygiene or cleanliness means), face washing practices (e.g., when adults and children wash their faces, what time of day is appropriate for face washing), the role of soap in face washing (e.g., when soap is necessary for face washing), and benefits to hygiene (e.g., advantages and benefits of general hygiene and face washing specifically). The focus groups lasted for approximately one hour and thirty minutes, and were conducted by a trained research staff member in Amharic. Focus groups were audio recorded, reviewed for identifying information, and transcribed verbatim. Transcripts were translated into English by a bilingual staff member and assessed for accuracy and consistency.

#### Quantitative phase

A quantitative survey was conducted with heads of households or their spouse in each of the five communities approximately 1 month after the focus group discussions. Study staff conducted a door-to-door household survey in each of the five communities in Amharic. Heads of households were interviewed about access to water, and questions about face washing and the use of soap. The questionnaire was asked to both male and female heads of household. If the head of household was not available the survey was conducted with the spouse. Questions were asked as multiple choice with an option for “other”, where a participant could provide an alternative response if none of the multiple-choice answers were applicable. Sociodemographic data collected included the age and sex of the respondent, the size of the family (total family size and number of children), and mobile phone ownership by any member of the household. Mobile phone ownership was included as a marker of socioeconomic status and reflects development in the region. Participants were asked about access to water, including how long water collection takes (round trip, dichotomized into less than 30 minutes versus greater than 30 minutes), and if the quantity of water the household receives from their primary water source is adequate for household needs. Interviewers also observed and recorded whether soap was available in the household at the time of the interview. Questions related to face washing and soap included whether or not the respondent had washed their face on the day of the interview, if all of the children in the household had washed their face on the day of the interview, if the household used soap, and if so, if soap was used for face washing.

Ethical approval for the study was obtained from the Committee for Human Research at the University of California, San Francisco and the Ethiopian Ministry of Science and Technology. Verbal informed consent in Amharic was obtained for all participants using an information sheet. The Institutional Review Board at each site granted approval for verbal informed consent. Verbal informed consent was obtained instead of written informed consent as the informed consent document would be the only document potentially identifying participants, the principle risk of the study was a breach of confidentiality, and because the study was judged to be no greater than minimal risk.

#### Analytic approach and data analysis

To obtain an understanding of community-level norms and perspectives of face washing knowledge, attitudes, and behaviors we employed focus group methodology. To ensure mixed-methods integration, focus group probes specifically linked to face washing, soap usage, and water access components of the quantitative survey and qualitative probes sought to elicit social discourse in order to understand collective and shared experiences. Using an immersion crystallization framework, we analyzed the transcripts based on an inductive and deductive approach to identify themes and relationships between themes and from each group (women, men, and community leaders). Codes were structured within specific overarching themes, including “face washing”, “hand washing”, and “latrines”. A list of codes was compiled each with a definition and example quote. The present analysis concentrated on codes under the “face washing” umbrella.

Three coders coded the transcripts. Inter-coder agreement was assessed via calculation of kappa coefficients via the code test application test in Dedoose Version 6.1.18 (SocioCultural Research Consultants, Los Angeles, CA; www.dedoose.com) in a randomly chosen transcript. Three tests with three separate transcripts were taken until kappa coefficients were at least 0.75 for each pair of coders (0.75, 0.81, and 0.88). Discrepancies were compared and discussed between the three coders after each test. Coding was done in Dedoose.

Quantitative data were described for face washing barriers by community and by using soap for face washing (yes versus no) with proportions for categorical variables and medians and interquartile ranges (IQR) for continuous variables. Logistic regression models with standard errors accounting for clustering by community were used to assess factors associated with 1) all children in the household having washed their face on the day of the interview, as reported by the interview respondent, and 2) the interview respondent reporting that the household uses soap for face washing. Bivariate logistic regression models for each independent variable, including age and sex of the respondent, number of children in the household, mobile phone ownership by a member of the household, the household being within a 30 minutes’ walk of the household’s water source, and whether or not the family’s daily water collection is adequate for all household needs were built for each of the two outcome variables. A multivariable logistic regression model was then built for each outcome variable including all independent variables in the model regardless of their statistical significance on the bivariate analysis. As a sensitivity analysis, we modeled three levels for distance to water source in a multivariable logistic regression model: >90 minutes (reference category), 60–90 minutes, and <30 minutes to explore if there was a dose-response relationship between distance to water source and face-washing of children. A complete case analysis was used. All standard errors were clustered by community using a clustered bootstrap with 1,000 replications. All quantitative analyses were run in Stata 13.1 (StataCorp, College Station, TX).

## Results

Of 279 eligible households, 264 households had data available on face washing for children and 279 for using soap for face washing. 154 of the survey respondents were female and 123 were male. The majority of heads of household were male (211); 66 households had a female head-of-household. Median age of the respondent in the household survey was 36 years (IQR 30 to 50). The majority of heads of households (98%) were farmers, and the median number of children in each household was 3 (IQR 2 to 4). A total of 105 individuals participated in 15 focus groups. Focus group participants ranged in age from 18 to 60 years, with a mean age of 35 (Table 1). The majority of focus group participants had no formal education.

**Table 1:** Focus group participant characteristics, stratified by community.

	Community				
	A	B	C	D	E
**Women**					
Mean age (range)	34 (30–36)	26 (18–30)	32 (28–60)	37 (25–45)	33 (24–47)
	SD 2.2	SD 5.4	SD 6.5	SD 8.3	SD 7.7
Education					
None	6 (86%)	6 (86%)	5 (71%)	7 (100%)	6 (86%)
Primary	1 (14)	1 (14%)	2 (29%)	0	4 (11%)
Secondary	0	0	0	0	1(3%)
**Men**					
Mean age (range)	43 (30–54)	38 (25–56)	39 (28–60)	35 (19–56)	35 (20–70)
	SD 10.1	SD 11.0	SD 14.0	SD 14.4	SD 17.0
Education					
None	2 (29%)	3 (43%)	5 (71%)	3 (43%)	2 (29%)
Primary	5 (71%)	5 (57%)	2 (29%)	3 (43%)	5 (71%)
Secondary	0	0	0	1 (14%)	0
**Community Leaders**					
Mean age (range)	32(18–45)	36 (18–58)	43 (32–65	37 (25–48)	37 (19–50)
	SD 10.4	SD 13.1	SD 11.5	SD 8.9	SD 11.0
Education					
None	3 (43%)	5 (71%)	3 (43%)	0	2 (29%)
Primary	4 (57%)	2 (29%)	4 (57%)	7 (100%)	5 (71%)
Secondary	0	0	0	0	0
Sex					
Female	4 (57%)	4 (57%)	0	0	4 (57%)
Male	3 (43%)	3 (43%)	7 (100%)	5 (71%)	3 (43%)

### Face washing knowledge, attitudes, and behaviors

Universally, participants endorsed daily face washing of their children, typically in the morning, although many participants also indicated that more frequent washing would be beneficial for their children.

#### Qualitative

Across focus groups, participant narratives from all communities and groups indicated that they washed their children’s face every day, and typically that washing was best done in the morning.

“We wash them in the morning when they wake up. We wash their hands and faces after they are done eating their breakfast to avoid flies immediately.”

(Women, Community E).

In the quantitative survey, nearly all (94%) respondents reported that they had washed their own face that day. Similar to face washing behaviors with children, respondents in the focus groups generally indicated that face washing was especially important in the morning.

“I myself wash [my face] in the morning; I am free from any dirty things. This means I will be free from any eye boogers on my face.”

(Community leaders, Community D)

Knowledge of the benefits of face washing for children was high across all groups and communities. Face washing was endorsed as a component of the general definition of hygiene.

“*We call a child clean when he washes his face after waking up*, *when he comes out of the toilet*, *when washing with soap and the like*, *and wears a clean cloth*.*”* (Community leaders, Community D)

Knowledge that face washing is beneficial for eye health in general as well as trachoma specifically was high across communities, especially among the community leaders and all-male focus groups.

“In the morning [face washing] is good because it would save his eyes from getting crossed by the touch of dirty hands. Hence, if an eye is washed properly, it means it would see clearly.”

(Men, Community B)

“We have to wash our children’s face in the morning, at noon, and in the evening in order to protect our eyes from trachoma.”

(Community leaders, Community B)

A commonly reported motivation for face washing for both children and adults was cleanliness and removal of dirt. Respondents also reported that face washing made them feel good, and thus was a motivator for their own face washing.

“We are farmers. We sweat when we work. We feel good when we wash and be clean. In addition, we wash our faces to avoid dirt that flies bring.”

(Women, Community E)

“When I wash in the morning, I feel refreshed. It has a very good advantage.”

(Men, Community C)

#### Quantitative

In the household survey, nearly all (92%; Table 2) participants indicated that all children in the household had washed their face that day, ranging from 86% in Community C to 100% in Community B and D. In multivariable models there was an association between proximity of the water source and the quantity of water collected being adequate for household needs and increased odds of all children washing their faces (Table 3). Households that lived within a 30-minute walk round trip of a water source, had 4.58 (95% CI 2.34 to 8.89, *P*<0.001) times higher odds of washing the faces of all children in the household compared to households that lived more than a 30 minute walk round trip from a water source. In addition, there was a dose-response relationship between distance to water source and washing the faces of children, with houses <30 minutes from the water source having 10.82 times the odds (95% CI 9.00 to 13.01, *P*<0.001), and those 60–90 minutes having 3.39 times the odds (95% CI 2.33 to 4.92, *P*<0.001) of face washing. Households that rated their quantity of water as adequate were also more likely to wash the faces of all children in the household compared to households that rated their water quality as inadequate (OR 2.86; 95% CI 1.35 to 6.03; *P* = 0.006). Mobile phone ownership, number of children in the household, and gender and age of respondent were not associated with all children in the household washing their faces.

**Table 2:** Self-reported household face washing behaviors, stratified by community.

	Community				
	A (N = 38 households)	B (N = 33 households)	C (N = 90 households)	D (N = 45 households)	E (N = 73 households)
Respondent washed face today	81.6%	100%	92.2%	100%	95.5%
All children in household washed face today	90.6%	100%	85.7%	100%	92.7%
Household uses soap for face washing	50.0%	51.5%	4.4%	77.8%	82.2%
Soap available in the household	50.0%	57.6%	4.4%	46.7%	91.7%

**Table 3:** Factors associated with self-report of all children in the household face washing on day of interview.

	Median (IQR) or N (%)					
	All children washed faced	Not all children washed face	Bivariate		Multivariable	
	(N = 242 households)	(N = 20 households)	*OR (95% CI)*^*1*^	*P-value*	*aOR (95% CI)*^*2*^	*P-value*
Age of respondent [median, IQR]	36 (30 to 50)	34.5 (30 to 66.5)	0.97 (0.92 to 1.04)	0.41	0.98 (0.90 to 1.06)	0.58
Female respondent	135 (56.3%)	10 (50.0%)	1.29 (0.79 to 2.10)	0.31	0.85 (0.52 to 1.40)	0.53
Number of children in household	3 (2 to 4)	2 (2 to 3)	1.43 (0.87 to 2.33)	0.16	1.42 (0.53 to 3.80)	0.48
Household mobile phone ownership	27 (11.2%)	3 (15.0%)	0.71 (0.27 to 1.89)	0.50	0.76 (0.04 to 13.18)	0.85
Water source within 30 minutes walk	141 (58.5%)	5 (25.0%)	4.23 (1.64 to 10.92)	0.003	4.58 (2.34 to 8.98)	<0.001
Quantity of water adequate for household needs	74 (37.6%)	2 (13.3%)	3.91 (1.00 to 15.27)	0.050	2.86 (1.35 to 6.03)	0.006

^1^Logistic regression model with standard errors estimated by bootstrap clustered by community

^2^Multivariable logistic regression model including all covariates in table with standard errors estimated by bootstrap clustered by community

Water-related barriers were not explicitly discussed specifically relating to face washing in the focus groups.

### Use of soap for face washing

Whereas there was universal endorsement of face washing among participants in quantitative and qualitative data, the reported usage of soap for face washing varied widely by community, from 4% in Community C to 82% in Community E (Table 2).

#### Qualitative

Despite low use of soap for face washing in some communities the quantitative survey, focus group participants in Community C reported knowledge of the benefits of using soap for face washing for adults and children.

“We apply soap in the morning in order to clean the discharge from our eyes.”

(Women, Community C)

“I call a child clean when he washes his hands and face with soap.”

(Community leaders, Community C)

However, women focus group members in Community C also noted that while they knew that soap was important for face washing, they often forgot to wash with soap.

“[Soap] is very crucial, but it becomes a habit to wash only with water and I tend to forget it.”

(Women, Community C)

Some focus groups participants noted that they only washed their face with soap in the morning, even if they endorsed face washing more frequently.

“Face washing with soap is necessary in the morning. Because in the morning our eye is usually dirty. After that, even if it is dusty soap is not necessary. Soap is necessary in the morning.”

(Women, Community A)

Cost was cited as a barrier to using soap for face washing by participants in two communities.

“There is no time that soap is not necessary unless we may not have soap since we are farmers… it’s just that we can’t afford it all the time.”

(Women, Community E)

*“Q*: *What do you think is the reason that you don’t use soap*? *A*: *It is because of the high living cost because one soap costs 7 or 8 Birr* [approximately USD$0.35].”

(Men, Community A)

#### Quantitative

The reported usage of soap for face washing varied widely by community, from 4% in Community C to 82% in Community E (Table 2). Mobile phone ownership, which is a marker of socioeconomic status, was associated with increased odds of using soap for face washing (aOR 3.19, 95% CI 1.13 to 9.01, *P* = 0.03; Table 4).

**Table 4:** Factors associated with self-report of household using soap for face washing.

	Median (IQR) or N (%)					
	Used soap for face washing	Did not use soap for face washing	Bivariate		Multivariable	
	(N = 135 households)	(N = 144 households)	*OR (95% CI)*^*1*^	*P-value*	*aOR (95% CI)*^*2*^	*P-value*
Age of respondent	35 (28 to 47)	38 (30 to 55)	0.98 (0.96 to 0.999)	0.04	0.98 (0.96 to 1.00)	0.12
Female respondent	96 (71.6%)	58 (50.6%)	3.70 (1.25 to 11.00)	0.02	1.83 (0.67 to 4.98)	0.24
Number of children in household	3 (2 to 5)	3 (2 to 4)	1.17 (1.04 to 1.31)	0.008	1.18 (1.07 to 1.29)	<0.001
Household mobile phone ownership	21 (15.7%)	9 (6.3%)	2.79 (1.61 to 4.81)	<0.001	3.19 (1.13 to 9.01)	0.03
Water source is within 30 minutes’ walk	98 (72.6%)	56 (39.2%)	4.11 (0.62 to 27.32)	0.14	2.73 (0.32 to 23.61)	0.36
Quantity of water adequate for household needs	53 (44.2%)	28 (26.9%)	2.15 (0.28 to 16.41)	0.46	1.81 (0.21 to 15.41)	0.59

^1^Logistic regression model with standard errors estimated by bootstrap clustered by community;

^2^Multivariable logistic regression model including all covariates in table with standard errors estimated by bootstrap clustered by community

### Face washing for prevention of flies

To gain a deeper understanding of face washing behaviors, focus groups explicitly probed for reasons behind face washing. Participants in all communities mentioned fly control as a reason for face washing, especially for children. Participants noted both that face washing was necessary when flies were seen on children’s faces, but also that regular face washing prevented flies from landing on faces.

“If I see flies on my children’s eyes then I get that they have not maintained proper hygiene… Because you have kept your hygiene flies do not swarm you. But since we have not maintained our cleanliness they swarm us.”

(Community leaders, Community C)

“For the flies not to lay around their eyes, they have to wash their faces otherwise their faces will be surrounded by flies.”

(Women, Community D)

Many participants cited trachoma prevention as a reason for face washing for fly control. The benefits of fly control were seen as beneficial for the general health and wellbeing of children, as well.

“First they will wash their faces, as a result flies won’t sit on them… Moreover, if we don’t take away dirt, flies will feed on it and lie on our children and transmit trachoma and other diseases. Our children will grow up properly.”

(Men, Community C)

## Discussion

In this mixed methods study, we document high prevalence of reported daily face washing among a rural population in a hyperendemic area for trachoma in Ethiopia. However, despite face washing being a common practice, soap was less commonly used as part of face washing routines. Face washing is a key component of the SAFE strategy for trachoma prevention, and use of soap may improve the ability of face washing to prevent trachoma transmission.[17,18] The use of soap for face washing in this study varied widely by community. Previous studies have demonstrated clustering of active trachoma and trachoma infection at the household and village level.[21,22] Geographic clustering of trachoma is likely due to both increased probability of transmission in areas with higher trachoma prevalence as well as shared characteristics such as environmental or climate factors.[23,24] The results of the present study suggest that there may be shared behavioral characteristics within villages that may also contribute to geographic clustering.

Although use of soap varied widely by community, focus group participants from all communities reported high levels of knowledge of the importance of soap. The focus groups suggested that economic barriers are important in limiting the regular use of soap for face washing, indicating economic interventions may be important for improving face washing with soap behaviors. In Community C, which had the lowest reported use of soap for face washing, focus group participants explained that while they knew the benefits of using soap for face washing, they were simply not in the habit of doing so. These results suggest that, in this community, individuals are further along the knowledge-attitude-behavior continuum in terms of behavior change.[25] As such, interventions promoting face washing with soap may be more effective if they focus on habit formation and practice rather than improving knowledge, as the community members already have knowledge of the benefits of using soap for face washing.

There is conflicting evidence of the relationship between distance to water source and trachoma.[16,26] Theoretically, increased distance to water source may reduce face washing behaviors because of water security in households. It is possible, however, that the inclusion of other factors in multivariable models (such as hygiene practices) obscures the relationship in some studies. In this study, we found an association between shorter distance to water source and face washing of children in the household. In addition, we noted a dose-response relationship, with households that reported longer times for water collecting less frequently reporting face-washing children. Similarly, households in which the survey respondent reported that the household had an adequate supply of water for their needs more often reported face washing of all children. The results indicate that face-washing behaviors may be facilitated by access to adequate water supply. Future work should consider the role of distance to water source on hygiene behaviors, as it is plausible that households with greater access to water have differential hygiene behaviors.

Overall in this sample, participants had high health literacy related to trachoma. Focus group participants generally believed that face washing would help prevent trachoma. The communities in which this study was conducted were in a region that is hyperendemic for trachoma, and as such received mass drug administrations for trachoma and participated in trachoma trials for the eight years prior to the present study.[27–29] High levels of trachoma knowledge may be related to participants’ involvement in these studies. This knowledge of trachoma and the fact that it could cause blindness in their children was likely a motivator for face washing behavior, explaining high coverage of daily face washing in this population. There is also the possibility that participants noted trachoma prevention as a motivation for face-washing because they felt it was the ‘correct’ answer and not because it was a true motivation. These results may not be generalizable to areas that are hyperendemic for trachoma that have not experienced this intensity of trachoma programming. Future work may be needed in trachoma study or program-naive populations to determine if face-washing predictors and behaviors differ.

The results of this study must be considered in the context of several limitations. Face washing behaviors in both the focus group discussions and the quantitative survey were collected via self-report. Although face washing is not necessarily a stigmatized behavior, it is possible that individuals’ responses may have been influenced by social desirability bias, as participants may have responded in ways which they perceived to be “correct”. There may have been alternative explanations, for example soap getting into children’s eyes, that were not discussed because participants perceived it was an incorrect answer. We anticipate that any outcome misclassification arising due to social desirability bias would be non-differential with respect to various predictors, and as such would, on average, bias towards the null in our regression models. In addition, while participants discussed their knowledge and current behaviors related to face washing and using soap, responses may not necessarily reflect motivations, and may rather reflect rationalization or normative reasons. Importantly, as qualitative data were collected as focus group discussions, individual responses may have been influenced by the responses of other members in the focus group. These results therefore should not be interpreted on the individual level, but instead represent community-level knowledge and behaviors. It is possible that some individual behaviors were masked in group discussions if some individuals did not want to discuss behaviors that were outlying from the rest of the group. Future work with individual interviews may yield additional insights into face washing and other hygiene behaviors in this region.

This study provides important insights into face washing knowledge, attitudes, and behaviors for intervention development in a trachoma hyperendemic region of rural Ethiopia. Overall knowledge of the benefits of face washing was high, and the use of soap for face washing varied widely by community. Water access was associated with reduced odds of all children in the household washing their faces, but was not discussed during focus group discussions. Barriers to face washing with soap included cost and forgetting to use soap. Interventions for face washing that include habit formation, which may help to address forgetfulness, and address structural barriers to accessing soap, like cost, may be important for increasing facial cleanliness and ultimately trachoma control in hyperendemic regions.

## Supporting Information

S1 STROBE Checklist (DOC)
